# COVID-19 and chronological aging: senolytics and other anti-aging drugs for the treatment or prevention of corona virus infection?

**DOI:** 10.18632/aging.103001

**Published:** 2020-03-30

**Authors:** Camillo Sargiacomo, Federica Sotgia, Michael P. Lisanti

**Affiliations:** 1 Translational Medicine, School of Science, Engineering and Environment (SEE), University of Salford, Greater Manchester, United Kingdom

**Keywords:** COVID-19, corona virus, aging, senescence, senolytic drug therapy, prevention, viral replication, drug repurposing, antibiotic, Azithromycin, Hydroxy-chloroquine, Rapamycin, Doxycycline, Quercetin

## Abstract

COVID-19, also known as SARS-CoV-2, is a new emerging zoonotic corona virus of the SARS (Severe Acute Respiratory Syndrome) and the MERS (Middle East Respiratory Syndrome) family. COVID-19 originated in China and spread world-wide, resulting in the pandemic of 2020. For some reason, COVID-19 shows a considerably higher mortality rate in patients with advanced chronological age. This begs the question as to whether there is a functional association between COVID-19 infection and the process of chronological aging. Two host receptors have been proposed for COVID-19. One is CD26 and the other is ACE-2 (angiotensin-converting enzyme 2). Interestingly, both CD26 and the angiotensin system show associations with senescence. Similarly, two proposed therapeutics for the treatment of COVID-19 infection are Azithromycin and Quercetin, both drugs with significant senolytic activity. Also, Chloroquine-related compounds inhibit the induction of the well-known senescence marker, Beta-galactosidase. Other anti-aging drugs should also be considered, such as Rapamycin and Doxycycline, as they behave as inhibitors of protein synthesis, blocking both SASP and viral replication. Therefore, we wish to speculate that the fight against COVID-19 disease should involve testing the hypothesis that senolytics and other anti-aging drugs may have a prominent role in preventing the transmission of the virus, as well as aid in its treatment. Thus, we propose that new clinical trials may be warranted, as several senolytic and anti-aging therapeutics are existing FDA-approved drugs, with excellent safety profiles, and would be readily available for drug repurposing efforts. As Azithromycin and Doxycycline are both commonly used antibiotics that inhibit viral replication and IL-6 production, we may want to consider this general class of antibiotics that functionally inhibits cellular protein synthesis as a side-effect, for the treatment and prevention of COVID-19 disease.

The earliest retrospective study of the COVID-19 outbreak in Wuhan, China, published in the Lancet, was among one of the first clinical studies to identify older age as a significant risk factor for in-hospital mortality, suggesting that advanced chronological age may play an epidemiological role in patient clinical outcomes [1].

Mortality was also associated with other co-morbidities, normally considered to be aging-associated diseases, such as diabetes or coronary heart disease, as well as a critical inflammatory mediator of the senescence-associated secretory phenotype (SASP), namely IL-6 [1].

This specific association of COVID-19 fatality with advanced chronological age was directly validated by the CDC in the US population [2] and published in the Morbidity and Mortality Weekly Report (MMWR) on the 18^th^ of March, as follows: “This first preliminary description of outcomes among patients with COVID-19 in the United States indicates that fatality was highest in persons aged ≥85, ranging from 10% to 27%, followed by 3% to 11% among persons aged 65–84 years, 1% to 3% among persons aged 55-64 years, <1% among persons aged 20–54 years, and no fatalities among persons aged ≤19 years”.

## What could be the biological mechanism(s) by which the COVID-19 virus preferentially targets patients with advanced chronological age?

Two host receptors have been proposed for COVID-19. One is CD26 [3] and the other is ACE-2 (angiotensin-converting enzyme 2) [4]. Interestingly, both CD26 and the angiotensin system show associations with senescence. For example, ACE-2 is a known inhibitor of cell proliferation and the angiotensin system is upregulated in both premature and replicative senescence [5,6]. Remarkably, CD26 is known to be a *bonafide* cell surface marker of senescent cells [7]. Similarly, myofibroblasts (which are considered to be senescent and pro-fibrotic cells) also over-express CD26 and ACE-2 [8,9]. Senescent cells produce large amounts of inflammatory cytokines, as a result of the senescence-associated secretory phenotype (SASP), including IL-6.

Interestingly, the host receptor for MERS-CoV, a highly-related corona virus, is CD26, also known as dipeptidyl-peptidase IV (DPP4) [10–12]. Genetic evidence, including functional studies of existing CD26 human polymorphisms and humanized CD26 transgenic mouse animal models, has directly shown that CD26 is the functional host receptor for MERS-CoV, which is specifically required for host cell attachment, entry and, therefore, productive host cell infections, as well as species restrictions [10–12] Moreover, recent structural studies predict that the COVID-19 spike glycoproteins also directly interact with host cell CD26 [3].

Thus, one hypothesis is that the COVID-19 virus significantly increases mortality in patients with advanced chronological age, because these patients have an increased number of senescent lung cells, which are the host target for COVID-19 viral infection. Interestingly, senescent cells also show an increased propensity for enhanced protein synthesis, which is required to produce SASP inflammatory mediators, which would make senescent cells an ideal host target for efficient viral replication.

Therefore, it would be predicted that senolytic drugs could have a beneficial effect for the treatment and/or prevention of COVID-19 disease. Is there any evidence to support this attractive hypothesis?

Recently, a clinical trial was conducted using COVID-19 positive hospitalized patients, which assessed COVID-19 virus production in response to treatment with two FDA-approved drugs, namely Hydroxy-chloroquine (Plaquenil) and Azithromycin (Z-PAC) [13]. Hydroxy-chloroquine alone, at the standard dosages, was surprisingly effective in reducing COVID-19 viral production. However, the combination of Hydroxy-chloroquine and Azithromycin appeared to be even more effective. The mechanism(s) by which this drug combination halts COVID-19 virus production remains unknown.

## What is the known relationship between Hydroxy-chloroquine, Azithromycin and senescence?

Chloroquine and its derivatives, such as Hydroxy-chloroquine, alkalinize the pH in lysosomes, which accumulate in large numbers in senescent cells. This Chloroquine-induced alkalinization functionally prevents the induction and accumulation of one of the most widely-recognized markers of senescence, known as beta-galactosidase (Beta-Gal), a lysosomal enzyme [14]. Hydroxy-chloroquine is also used clinically for the treatment of chronic inflammatory diseases, such as Sjögren's syndrome, and it effectively reduces the salivary and serum levels of IL-6, a key component of the SASP [15].

Azithromycin also has a key relationship with senescence [16]. Recent studies have shown that Azithromycin, and the closely related drug Roxithromycin, both act as senolytic drugs that can target and selectively remove senescent cells, with an efficiency of nearly 97% [16]. Interestingly, in patients with Cystic Fibrosis, Azithromycin is known to have an anti-fibrotic effect, which significantly extends their lifespan, by targeting myofibroblast cells (Discussed in Ref [16]). Cystic Fibrosis patients normally die from lung inflammation and fibrosis, resulting in lung stiffening and an inability to respire. Fibrosis is also known to be an age-related phenomenon, associated with increased numbers of myofibroblasts (senescent cells), which increases with chronological age. Azithromycin functionally acts as an anti-inflammatory drug and reduces SASP mediators, such as IL-1beta and IL-6 [17,18]. This may be due to Azithromycin’s high senolytic activity and/or inhibition of protein synthesis.

Interestingly, Azithromycin also inhibits the replication of other viruses, such as Zika and Ebola [19–21]. If this inhibitory activity reflects Azithromycin’s ability to inhibit protein synthesis, then other inhibitors of protein synthesis, such as Rapamycin, should be considered as well (Supplementary Figure 1).

Consistent with this hypothesis, Rapamycin has been shown to potently inhibit HIV-1 replication [22]. Moreover, Rapamycin shows key anti-aging properties and prevents the onset of senescence [23–25].

Similarly, Doxycycline inhibits mammalian cell protein synthesis as an off-target side effect [26], effectively blocks replication of Dengue virus [27], reduces IL-6 serum levels during viral infection [28] and behaves as an anti-aging drug [29]. Therefore, Doxycycline could provide another inexpensive, but very attractive, option for the treatment or prevention of COVID-19 infection.

Finally, a recent study, using supercomputer-based *in silico* drug-docking to the COVID-19 viral spike protein identified Quercetin as a potential binding partner, to reduce virus-host interactions, with ACE-2 [30]. Quercetin has also been identified as a dietary supplement with senolytic properties [31].

**Figure 1:**
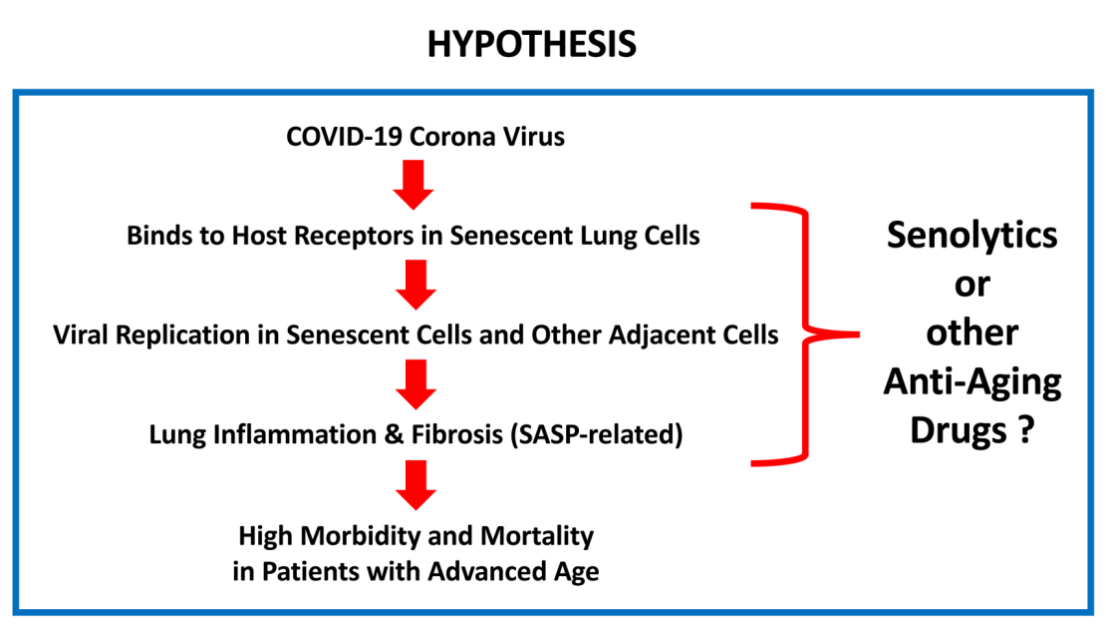
**What is the relationship between COVID-19 and advanced chronological age?** Here, we suggest that the COVID-19 corona virus preferentially targets senescent lung cells, resulting in increased morbidity and mortality in the aging population. One possible solution for prevention/treatment would be the use of senolytics or other anti-aging drugs. Testing this hypothesis will require the necessary clinical trials, with a focus on drug repurposing.

Therefore, we propose that the clinical relationship between advanced chronological age and COVID-19 mortality may suggest the use of senolytic or anti-aging drugs in COVID-19 disease prevention (Figure 1). Of course, clinical trials will be necessary to test this attractive, but speculative, hypothesis experimentally.

Fortunately, several promising senolytic and other anti-aging drugs are already FDA-approved for other disease indications. This approach can significantly accelerate their clinical evaluation through drug repurposing, as they have already been evaluated for their clinical safety, in Phase I trials. As such, these FDA-approved drugs can directly enter into Phase II clinical trials, to test their potential efficacy against COVID-19. Alternatively, in the United States, FDA-approved drugs can be medically-prescribed for an “off-label” use, at the discretion of the practicing physician.

Interestingly, SARS-CoV, a close relative of COVID-19 (SARS-CoV-2), also shows increased susceptibility in patients with advanced chronological age, which has been recapitulated in a mouse animal model of disease pathogenesis [32,33]. Briefly, in young mice (4-8 weeks-old), the SARS-CoV infection is cleared very rapidly, which is accompanied by mild pneumonitis, without the activation of cytokine production. In contrast, in older mice (12-14-months-old), productive infection with SARS-CoV led to a more severe interstitial pneumonitis, with alveolar damage, significant fibrosis and scarring, as well as severe activation of cytokine production, including TNF-α, IL-6, CCL-2, CCL-3, CXCL-10, and IFN-γ [32,33]. This latter mouse model more closely resembles the SARS-CoV disease phenotype, observed in patients with advanced chronological age. Therefore, such a mouse model would also be useful for testing the efficacy of new therapies, specifically targeting the senescent cell population and SASP, for the repurposing of these FDA-approved anti-aging drugs.

## Disclaimer

This perspective is intended for a professional audience, to stimulate new ideas and to aid the global efforts to develop effective treatments for COVID-19 disease. This article does not represent medical advice or recommendations to patients. The media should exercise caution and seek expert medical advice for interpretation, when referring to this article.

## Note Added in Proof

While this Perspective was being reviewed, another clinical trial was published online, confirming the efficacy of Hydroxy-chloroquine and Azithromycin, for treating COVID-19 patients, in a larger cohort of 80 patients. See the following article:

Philippe Gautret, Jean-Christophe Lagier, Philippe Parola, Van Thuan Hoang, Line Meddeb, Jacques Sevestre, Morgane Mailhe, Barbara Doudier, Camille Aubry, Sophie Amrane, Piseth Seng, Marie Hocquart, Julie Finance, Vera Esteves Vieira, Hervé Tissot Dupont, Stéphane Honoré, Andreas Stein, Matthieu Million, Philippe Colson, Bernard La Scola, Véronique Veit, Alexis Jacquier, Jean-Claude Deharo, Michel Drancourt and Didier Raoult. Clinical and microbiological effect of a combination of hydroxychloroquine and azithromycin in 80 COVID-19 patients with at least a six-day follow up: an observational study. https://www.mediterranee-infection.com/wp-content/uploads/2020/03/COVID-IHU-2-1.pdf

## Supplementary Material

Supplementary Figure
