# Supplementary material for: COVID-19 and chronological aging: senolytics and other anti-aging drugs for the treatment or prevention of corona virus infection?
Source: Aging (Albany NY). 2020 Mar 30;12(8):6511–7. doi: 10.18632/aging.103001 (PMC7202514; doi:10.18632/aging.103001)
Supplement: Supplementary Figure [file aging-12-103001-s001..pdf]

SUPPLEMENTARY FIGURE

Cytokines and Viruses are both made of Proteins

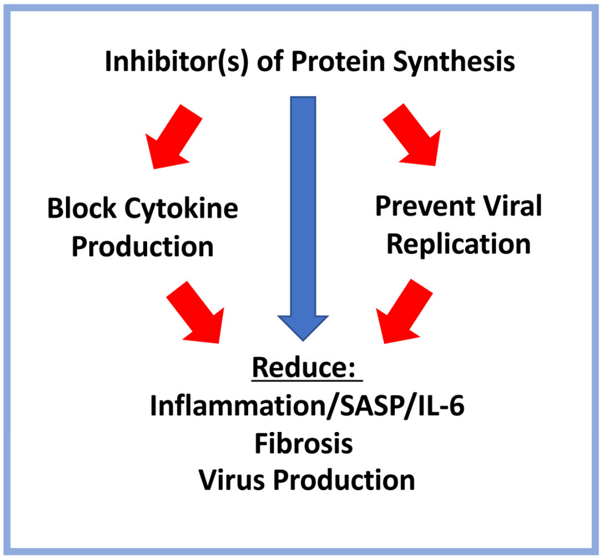

Doxycycline, Azithromycin and Rapamycin All Inhibit Protein Synthesis
All are FDA-Approved Drugs

Supplementary Figure 1. Inhibitor(s) of protein synthesis block inflammation and viral replication. Azithromycin, Doxycycline and Rapamycin are all FDA-approved drugs that behave as inhibitors of protein synthesis and experimentally have been shown to reduce inflammation and viral replication. Mechanistically, this is because cytokines and viruses are both made of proteins. Both use the cellular ribosomes for protein translation. Inhibiting virus production should help to clinically reduce viral transmission to other patients.
